# Outcomes and Complications of Aggressive Resection Strategy for Pituitary Adenomas in Knosp Grade 4 With Transsphenoidal Endoscopy

**DOI:** 10.3389/fonc.2021.693063

**Published:** 2021-06-21

**Authors:** Taohui Ouyang, Na Zhang, Shenhao Xie, Bin Tang, Junjun Li, Limin Xiao, Fabao Zhang, Bowen Wu, Dongwei Zhou, Meihua Li, Tao Hong

**Affiliations:** ^1^ Department of Neurosurgery, the First Affiliated Hospital of Nanchang University, Nanchang, China; ^2^ Department of Neurology, the First Affiliated Hospital of Nanchang University, Nanchang, China; ^3^ Department of Neurosurgery, Wuhan Union Hospital, Wuhan, China; ^4^ Department of Radiology, the First Affiliated Hospital of Nanchang University, Nanchang, China

**Keywords:** endoscopic, cavernous sinus, pituitary adenoma, outcome, surgery

## Abstract

Surgery for pituitary adenomas (PAs) with cavernous sinus (CS) invasion in Knosp grade 4 is a great challenge and whether to adopt a conservative or aggressive surgical strategy is controversial. The aim of this study is to provide the outcomes and complications of an aggressive resection strategy for Knosp grade 4 PAs with transsphenoidal endoscopic surgery. Outcomes and complications were retrospectively analyzed in 102 patients with Knosp grade 4 PAs. Among them, primary PAs were seen in 60 patients and recurrent PAs were seen in 42 cases. Gross total resection (GTR) of the entire tumor was achieved in 72 cases (70.6%), subtotal tumor resection (STR) in 18 cases (17.6%), and partial tumor resection (PTR) in 12 cases (11.8%). Additionally, GTR of the tumor within the CS was achieved in 82 patients (80.4%), STR in 17 patients (16.7%), and PTR in 3 patients (2.9%). Statistical analyses showed that both recurrent tumors and firm consistency tumors were adverse factors for complete resection (P<0.05). Patients with GTR of the entire tumor were more likely to have favorable endocrine and visual outcomes than those with incomplete resection (P<0.05). Overall, the most common surgical complication was new cranial nerve palsy (n=7, 6.8%). The incidence of internal carotid artery (ICA) injury and postoperative cerebrospinal fluid (CSF) leakage was 2.0% (n=2) and 5.9% (n=6), respectively. Six patients (5.9%) experienced tumor recurrence postoperatively. For experienced neuroendoscopists, an aggressive tumor resection strategy *via* transsphenoidal endoscopic surgery may be an effective and safe option for Knosp grade 4 PAs.

## Introduction

Pituitary adenomas (PAs) are the third most common primary intracranial tumor, following meningiomas and gliomas, accounting for about 10% to 25% of intracranial tumors (1, 2). Some PAs invade surrounding structures such as the diaphragma sellae, the sphenoid sinus, and the cavernous sinus (CS), with approximately 10% involving the CS (3, 4). PAs invading the CS are particularly surgically challenging due to their close proximity to critical neurovascular structures and their deep intracranial location.

For PAs with CS invasion, surgical approaches include transcranial microscopy, transsphenoidal microscopy, and transsphenoidal endoscopy. Transcranial microscopy is a “lateral to medial” approach that basically entails entering from the lateral wall of the CS where multiple cranial nerves are located (5, 6). In addition, surgical visualization when utilizing the transcranial microscopy approach is often insufficient. These disadvantages support the argument that transcranial microscopic surgery is not an ideal approach for the removal of PAs with CS invasion. Since its introduction, the transsphenoidal approach has been continuously refined and popularized, and to date is the most common approach for PAs. Transsphenoidal approach is a “medial to lateral” approach that enters the CS through the medial CS wall, thereby avoiding direct obstruction by the cranial nerves within the lateral CS wall. Moreover, PAs invade the CS in a medial to lateral direction, often pushing the CS contents medially to laterally. Therefore, the transsphenoidal approach is an excellent and logical route for the removal of PAs within the CS through the medial CS wall. However, certain limitations such as a narrow surgical corridor and insufficient visualization, confine use of the transsphenoidal microscopic approach to those PAs with mild CS invasion (Knosp grades 1-2) (7). Compared with transsphenoidal microscopy, transsphenoidal endoscopic surgery not only improves surgical visualization, but also provides greater exposure to the lateral CS extension of tumors (7, 8). Consequently, transsphenoidal endoscopic surgery is the preferred choice for PAs with severe CS invasion (Knosp grades 3-4) (9, 10).

For PAs with severe CS invasion, especially grade 4 PAs, gross total resection (GTR) with transsphenoidal endoscopic surgery still presents a great challenge. To illustrate, Hwang et al. (11) retrospectively examined data on 275 patients with non-functional PAs with CS invasion from 2000-2012, and reported a GTR rate of 5.9% in transsphenoidal endoscopy for grade 4 PAs. This low GTR rate reflects the conservative strategy regarding tumor resection, but it also indicates that in some cases with grade 4 PAs, GTR can be achieved by transsphenoidal endoscopic surgery. Of note, these cases occurred a decade ago when transsphenoidal endoscopic technology was still in its infancy.

With the recent, rapid development in transsphenoidal endoscopic techniques and advances made through in-depth study of the endoscopic anatomy of the CS (12, 13), as well as the availability of intraoperative neuronavigation, doppler ultrasonography, and cranial nerve monitoring, it is worth reconsidering whether it is best to adopt a conservative or aggressive surgical strategy for grade 4 PAs. The current study provides the outcomes and complications of an aggressive surgical strategy for 102 patients with grade 4 PAs treated by transsphenoidal endoscopic surgery performed by the same surgeon.

## Materials and Methods

### Clinical Materials

Between January 2014 and August 2020, 908 consecutive patients undergoing transsphenoidal endoscopic surgery for PAs were evaluated. A total of 102 patients were included in this study according to the following criteria: 1) Knosp grade 4 PAs identified jointly by a neuroradiologist and two neurosurgeons; 2) Complete preoperative and postoperative magnetic resonance imaging (MRI) data and follow-up data; and 3) All operations performed by the same neurosurgeon (Tao Hong). A total of 4 cases were excluded due to lack of complete follow-up data. Hospital review board approval was obtained from the Institutional Ethics Committee of the First Affiliated Hospital of Nanchang University.

### Neuroradiological Evaluation

MRI examination was performed for all patients to evaluate tumor size and volume. Tumors with a maximum diameter (MD) of more than 1 cm were defined as macroadenomas, and tumors with a MD of more than 4 cm were defined as giant PAs. Tumor volume was approximated by calculations using the sphericity formula (A×B×C) ×π/6, where A, B and C represent the maximum length in three dimensions of the PA. Extent of resection for the entire tumor and tumors within the CS were analyzed and the extent of resection was then classified into categories: gross-total resection (GTR) (no residual enhancing lesion), subtotal tumor resection (STR) (residual enhancing lesion ≤ 20%) and partial tumor resection (PTR) (residual enhancing lesion >20%).

### Evaluation of the Internal Carotid Artery (ICA)

A preoperative balloon occlusion test was used to evaluate the contralateral compensatory capability of the ICA in case of injury. Although the anatomy of the ICA was generally consistent, the shape of the ICA under the influence of the tumor was inconsistent and individualized depending on the tumor location. Preoperatively, three-dimensional (axial, coronal, and sagittal) MRI was used to individually analyze the spatial relationship between the ICA and the CS tumor, and further, to determine the size of the space for each compartment within the CS. For example, when the horizontal ICA was inferior to the tumor, it caused the horizontal ICA to sink due to the depression of the tumor, resulting in a larger posterosuperior compartment and a smaller anteroinferior compartment within the CS. When the horizontal ICA was superior to the tumor, it caused the horizontal ICA to be raised by the tumor, leading to a smaller posterosuperior compartment and a larger anteroinferior compartment within the CS. When the horizontal ICA was in the middle of the tumor, the result was a combination of the above two conditions, leaving large posterosuperior and anteroinferior compartments.

### Endoscopic Surgery of PA in Knosp Grade 4

**Figure 1:**
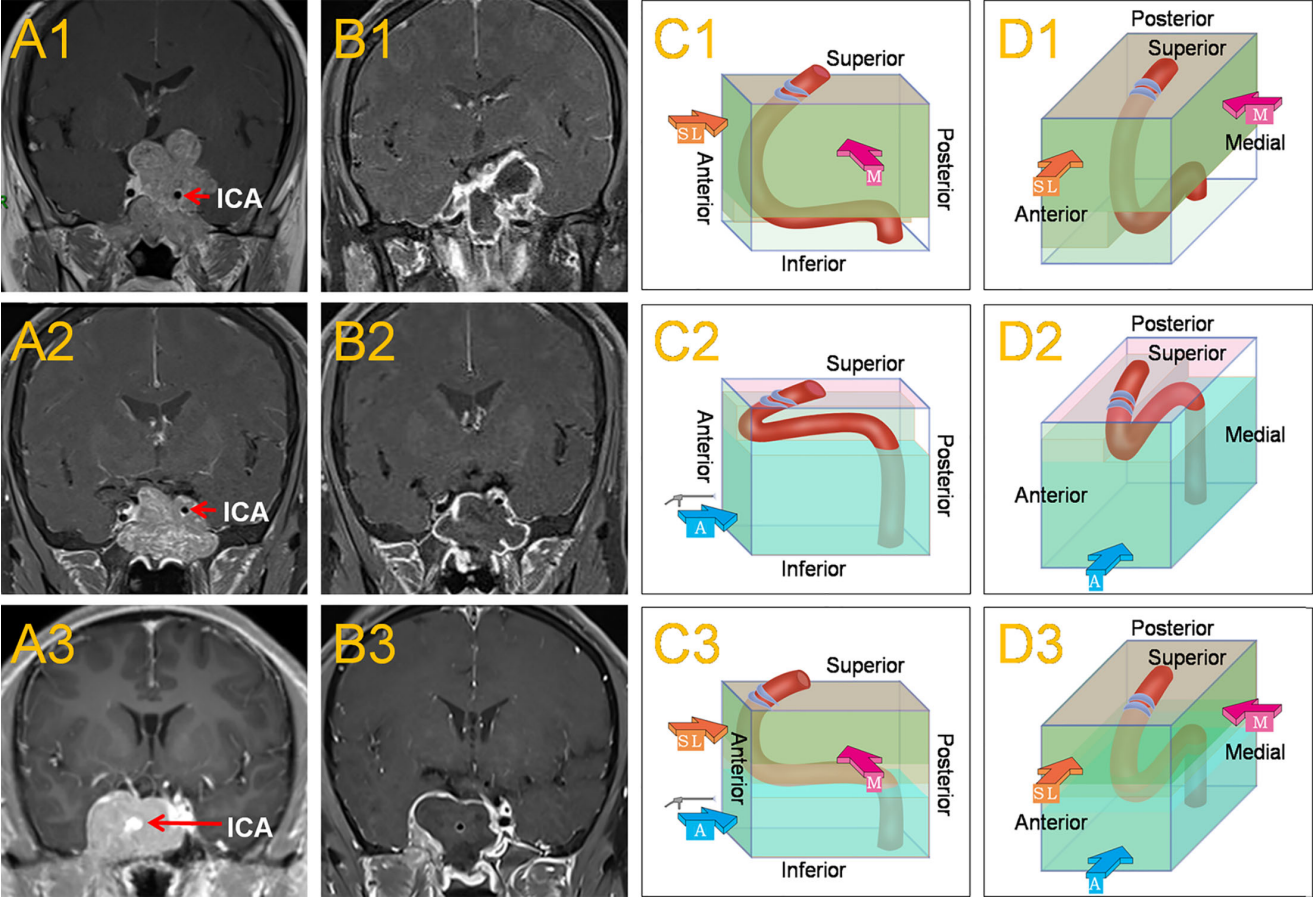
This shows the common location of the horizontal ICA in Knosp grade 4 PAs and the main surgical approaches. When the horizontal ICA was inferior to the tumor **(A1)**, it caused the horizontal ICA to sink due to the oppression of the tumor, resulting in a larger posterosuperior compartment and a smaller anteroinferior compartment within the CS. The medial approach, often combined with the superior-lateral approach was mainly used to remove the tumor **(C1, D1)**. Postoperative MRI revealed a subtotal resection of the tumor **(B1)**.When the horizontal ICA was superior to the tumor **(A2)**, it caused the horizontal ICA to be raised by the tumor, leading to a smaller posterosuperior compartment and a larger anteroinferior compartment within the CS. The anteroinferior approach was mainly used to remove the tumor **(C2, D2)**. Postoperative MRI revealed a total resection of the tumor **(B2)**. When the horizontal ICA was in the middle of the CS tumor **(A3)**, which was a combination of the two conditions, then the medial, superior-lateral, and anteroinferior approaches could be used **(C3, D3)**. Postoperative MRI revealed a total resection of the tumor **(B3)**. The red, thick arrow ‘M’ represents the medial approach, the orange-yellow, thick arrow ‘SL’ represents the superior-lateral approach, and the blue, thick arrow ‘A’ represents the anteroinferior approach.

For PAs invading the CS, transsphenoidal endoscopic surgery mainly included medial (transsphenoidal transsellar) and lateral (transmaxillary transpterygoidal) approaches. The medial approach was used to remove tumors in the posterosuperior and lateral compartments of the CS. The lateral approach, including an anteroinferior approach and a lateral-superior approach, was used to open the pterygopalatine fossa to obtain more exposure, and to remove tumors in the anteroinferior and lateral compartments of the CS. These approaches were shown in **Figure 1**. An angular endoscope was used to obtain different angles of surgical field visualization, and various other angular instruments were used to assist with the medial and lateral approaches. In many cases, a combination of medial and lateral approaches was required to radically remove the tumor. Intraoperative navigation and doppler ultrasonography were used to assist in determining the exact position and shape of the ICA. Before entering the CS to remove the tumor, the proximal end (paraclivus segment) of the ICA was exposed in order to temporarily block the proximal end in the event of injury to the ICA. Based on these analyses above, the individual spatial relationship among the ICA, the CS tumor, and various compartments of the CS were evaluated in order to determine the point of entry to the CS and the type of surgical approach to take within the CS. For example, when the horizontal ICA was inferior to the tumor, first, the medial wall of the CS was opened into the posterosuperior compartment of the CS, and then a combination of angle endoscopy and the medial approach was used to remove the tumor in the posterosuperior and lateral compartments. With this approach, the tumor extending into the oculomotor nerve triangle of the posterosuperior compartment might remain unresectable; in this case, a combination of the lateral-superior approach and the medial approach was required. When the horizontal ICA was superior to the tumor, first, the anterior wall of the CS was opened and then the anteroinferior approach was used to remove the tumor in the anteroinferior compartment and a lateral-superior approach was used for removing the tumor in the lateral compartment. When the horizontal ICA was in the middle of the CS tumor, which is a combination of the two conditions, then various approaches could be used. Particularly, in some special cases, such as when the ICA was medial to the tumor, which was common in recurrent tumors, a lateral approach was used to remove the tumor. When the ICA was lateral to the tumor, a medial approach alone was used. During tumor resection, any adhesion between the ICA and the abducens nerve requires sharp dissection to avoid injury to the abducens nerve. Care also needs to be taken to avoid injury to the oculomotor nerve in the removal of tumors within the posterosuperior compartment of the CS or even when extending to the temporal lobe through the oculomotor nerve triangle.

After tumor removal, the multilayered method of fat + artificial dura + fascia lata + pedicled mucosal flap + biological glue was used to reconstruct the sellar floor. For patients at high risk of cerebrospinal fluid (CSF) leakage, a lumbar puncture catheter drainage system was used to release CSF to prevent CSF leakage after surgery.

### Endocrinological Assessment

Endocrinological results of all the patients were evaluated preoperatively and at 3 months postoperatively. Hormone remission was based on the following criteria (14–17): for prolactin (PRL)-secreting adenomas, serum PRL <20 ng/mL in female patients or, <15 ng/mL in male patients; for adrenocorticotropic hormone (ACTH)-secreting adenomas, a serum cortisol nadir of < 2 mg/dL or normal 24-hour urinary free cortisol test at 3 months; and for growth hormone (GH)-secreting tumors, normalization of serum insulin-like growth factor-1 level (IGF-1), basal serum GH <2.5 ng/mL, or an oral glucose tolerance test of 0.4 ng/mL. During the test, IGF-1 levels were expressed relative to the upper limit of the patient’s normal values for the patient’s age and sex. The first choice for the treatment of PRL-secreting adenomas was drugs, and surgical indications were mainly drug-resistant tumors and drug intolerance, and rarely, patient choice.

### Visual Assessment

All patients received preoperative and postoperative formal visual function examinations. During follow-up, a visual examination was performed at 3 months, postoperatively.

### Statistical Analysis

Statistical analyses comparing categorical variables were performed using X^2^ tests or Fisher’s exact tests. A two-tailed *P*<0.05 was considered to be statistically significant. Data were analyzed using SPSS version 25.0 (IBM Corporation, Armonk, New York).

## Results

### Patient Population and Clinical Presentation

The 102 patients in the final analysis included 53 females and 49 males, of mean age 39.1 years (range 24-71 years). Primary PAs were seen in 60 patients and recurrent PAs were seen in 42 cases. Preoperative headache symptoms were seen in 33 patients (32.4%), visual dysfunction in 38 patients (37.3%), incidental discovery in 8 patients (7.8%), and cranial nerve palsy in 6 patients (5.9%). The detailed results are shown in **Table 1**.

**Table 1:** Demographic and clinical characteristics of the 102 patients with pituitary adenomas invading the CS.

Variables	Value
Mean age, years (range)	39.1 (24~71)
Females (%)	53 (52.0)
**Preoperative manifestation**	
High hormone level (%)	40 (39.0)
Headache (%)	33 (32.4)
Visual dysfunction (%)	38 (37.3)
Cranial nerve palsy (%)	6 (5.9)
Incidental discovery (%)	8 (7.8)
**Endocrinological types**	
Non-functional (%)	62 (60.8)
GH (%)	13 (12.7)
GH/PRL mixed (%)	10 (9.8)
PRL (%)	9 (8.8)
ACTH (%)	5 (4.9)
FSH (%)	2 (2.0)
TSH (%)	1 (1.0)
**Direction of invasion**	
Bilateral (%)	36 (35.3)
Unilateral (%)	66 (64.7)
Left (%)	35 (53.0)
Right (%)	31 (47.0)
**Contralateral (%)**	
Knosp 0 (%)	19 (28.8)
Knosp 1 (%)	17 (25.8)
Knosp 2 (%)	14 (21.2)
Knosp 3 (%)	16 (24.2)
**Size of tumor**	
Giant pituitary adenoma	39 (38.2)
Macroadenoma	63 (61.8)
**Volume of tumor (V, cm^3^)**	
V<15	22 (21.6)
15≤V ≤ 30	37 (36.3)
>30	43(42.1)
**Previous treatment**	
No surgery (%)	60 (58.8)
Craniotomy (%)	5 (4.9)
Transnasal microscopy (%)	12 (11.8)
Transnasal endoscopic (%)	15 (14.7)
Mixed surgery (%)	10 (9.8)
Radiotherapy (%)	16 (15.7)

### Neuroradiological Outcomes

Of the 102 cases, 39 patients had giant PAs and 63 patients had macroadenomas. The median tumor volume in all patients was 23.8 cm^3^, ranging from 1.63~182 cm^3^. In 36 patients, bilateral CS invasion was in grade 4, while unilateral CS invasion in grade 4 was observed in 66 patients (**Table 1**).

### Extent of Resection

**Figure 2:**
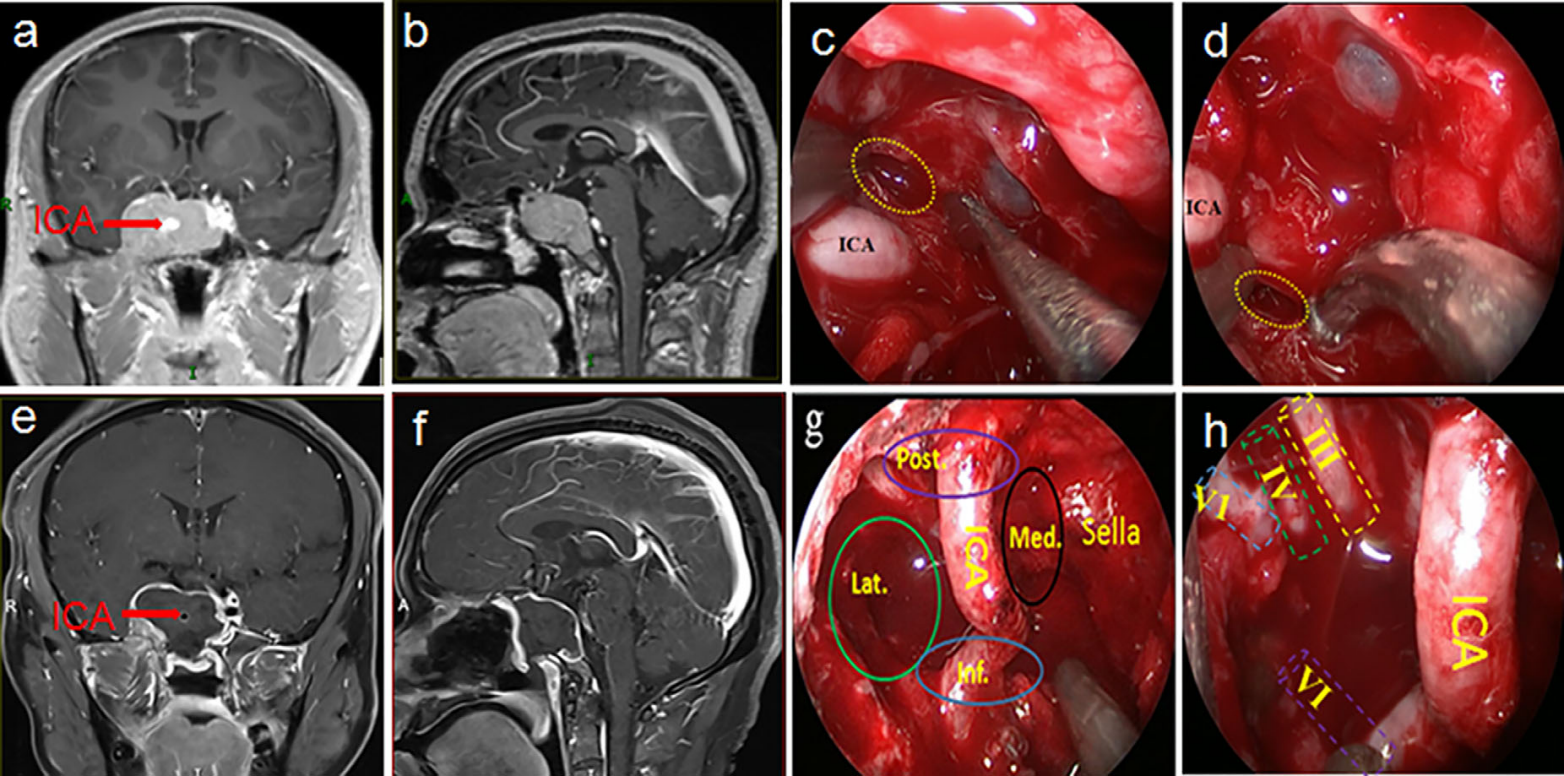
This was a 32-year-old male patient who was admitted to hospital after the accidental discovery of a pituitary tumor due to trauma. The preoperative visual examination was normal, and the preoperative PRL and ACTH hormones were increased. A preoperative MRI **(A, B)** showed that the tumor was in Knosp grade 4 and was mainly distributed in both posterosuperior and anteroinferior compartments of the CS. Intraoperatively, various compartments **(G)** and nerves **(H)** in the CS could be observed after total tumor resection. Postoperative MRI **(E, F)** also confirmed total tumor resection. The yellow dotted circle indicated entry into the CS from above **(C)** and below **(D)** the horizontal ICA. The pathological examination of the tumor was mixed pituitary adenoma. The visual and endocrine function of the patient were all normal three months after the operation. Post., posterior compartment; Med., medial compartment; Lat., lateral compartment; Inf., inferior compartment; ICA, internal carotid artery; III, oculomotor nerve; IV, trochlear nerve; V1, first branch of the trigeminal nerve; VI, abducens nerve.

GTR of the entire tumor was achieved in 72 cases (70.6%), STR in 18 cases (17.6%), and PTR in 12 cases (11.8%). **Figure 2** illustrated the GTR of the entire tumor. For tumors within the CS, GTR was achieved in 82 patients (80.4%), STR in 17 patients (16.7%), and PTR in 3 patients (2.9%). The GTR rates of the entire lesion and the CS lesion in the primary tumor were 80% and 91.7%, respectively, while the GTR rates of the entire lesion and the CS lesion in recurrent tumors were 57.1% and 64.3%, respectively. These results are shown in **Tables 2** and **3**. Statistical analyses showed that recurrent tumors and firm consistency tumors had lower GTR rates (p<0.05). However, no statistical differences were found between tumor size or tumor volume and the extent of resection (p>0.05). Additionally, there was no statistically significant relationship between extent of resection and postoperative CSF leakage or cranial nerve palsy (p>0.05). These results are shown in **Tables 3** and **4**.

**Table 2:** Surgical results and postoperative complications in the 102 patients who underwent transsphenoidal endoscopic surgery for pituitary adenomas in Knosp Grade 4.

Variable	Value
**Extent of resection**	
GTR (%)	72 (70.6)
STR (%)	18 (17.6)
PTR (%)	12 (11.8)
**Extent of CS resection**	
GTR (%)	82 (80.4)
STR (%)	17 (17.6)
PTR (%)	3 (2.0)
**Intraoprative variables**	
Mean operation time (min)	158.5
Mean intraoperative hemorrhage (ml)	456.7
Firm consistency (%)	31 (30.4)
ICA rupture (%)	2 (2.0)
CSF leakage (%)	11 (10.8)
**Postoperative variables**	
**Headache**	33
Better (%)	22 (66.7)
No change (%)	11 (33.3)
**Visual dysfunction**	38
Better (%)	28 (73.7)
No change (%)	10 (26.3)
**Hormone remission**	40
Yes (%)	29 (72.5)
No (%)	11 (27.5)
**Postoperative complications**	
abducens nerve palsy (%)	3 (2.9)
oculomotor nerve palsy (%)	4 (3.9)
CSF leakage (%)	6 (5.9)
CNS infection (%)	5 (4.9)
Monocular blindness (%)	2 (2.0)
Panhypopituitarism (%)	3 (2.9)
Permanent diabetes insipidus (%)	2 (2.0)
**Follow-up treatments**	
Radiotherapy (%)	12 (11.8)
Medical therapy (%)	5 (4.9)
Recurrence (%)	6 (5.9)

GTR, gross total resection; STR, subtotal tumor resection; PTR, partial tumor resection; CS, cavernous sinus; CSF, cerebrospinal fluid; CNS, Central Nervous System; ICA, Internal carotid artery.

**Table 3:** Surgical results and postoperative complications stratified by primary and recurrent pituitary adenomas.

Variables	Primary	Recurrent	P value
No. of patients (%)	60 (58.8)	42 (41.2)	
**Extent of resection**			
GTR (%)	48 (80.0)	24( 57.1)	**0.013**
STR (%)	8 (13.3)	10 (23.8)	0.172
PTR (%)	4 (6.7)	8 (19.1)	0.056
**Extent of CS resection**			
GTR (%)	55 (91.7)	27 (64.3)	**0.001**
STR (%)	4 (6.7)	13 (31.0)	**0.001**
PTR (%)	1 (1.6)	2 (4.7)	0.363
**Intraoprative variables**			
ICA rupture (%)	0 (0.0)	2 (4.8)	0.088
CSF leakage (%)	4 (6.7)	7 (16.7)	0.109
Firm consistency (%)	14 (23.3)	17 (40.5)	0.064
**Postoperative complications**			
CSF leakage (%)	2 (3.3)	4 (9.5)	0.191
CNS infection (%)	2 (3.3)	3 (7.1)	0.380
Monocular blindness (%)	1 (1.7)	1 (2.4)	0.798
Panhypopituitarism (%)	1 (1.7)	2 (4.8)	0.363
Permanent diabetes insipidus (%)	1 (1.7)	1 (2.4)	0.798
**Follow-up treatments**			
Radiation therapy (%)	5 (8.3)	7 (16.7)	0.199
Medical therapy (%)	2 (33.3)	3 (7.1)	0.380

CS, cavernous sinus; CSF, cerebrospinal fluid; GTR, gross total resection; STR, subtotal tumor resection; PTR, partial tumor resection; CNS, central nervous system; ICA: internal carotid artery. In bold: represent statistical significance.

**Table 4:** Extent of resection correlated with multiple potential variables.

Variables	Within the CS			Entire tumor		
	GTR of CS	STR of CS	P value	GTR	STR	P value
**Size of tumor**						
Giant PA	31 (37.8)	7 (41.2)		28 (38.9)	9 (50.0)	
Macroadenoma	51 (62.2)	10 (58.8)	0.795	44 (61.1)	9 (50.0)	0.391
**Volume of tumor(V, cm^3^)**						
V<15	19 (23.2)	3 (17.6)		16 (22.2)	4 (22.2)	
15≤V ≤ 30	32 (39.0)	4 (23.5)		29 (40.3)	6 (33.3)	
>30	31 (37.8)	10 (58.9)	0.269	27 (37.5)	8 (44.5)	0.836
**Firm consistency**						
Yes	19 (23.2)	12 (70.6)		16 (22.2)	10 (55.6)	
No	63 (76.8)	5 (29.4)	**<0.001**	56 (77.8)	8 (44.4)	**0.005**
**Postoperative CSF leak**						
Yes	4 (4.9)	2 (11.8)		3 (4.2)	3 (16.7)	
No	78 (95.1)	15 (88.2)	0.279	69 (95.8)	15 (83.3)	0.057
**Postoperative cranial nerve palsy**						
Yes	4 (4.9)	3 (17.6)		4 (5.6)	3 (16.7)	
No	78 (95.1)	14 (82.4)	0.062	68 (94.4)	15 (83.3)	0.115

PA, pituitary adenoma; CS, cavernous sinus; CSF, cerebrospinal fluid; GTR, gross total resection; STR, subtotal tumor resection. In bold: represent statistical significance.

### Surgical Complications

Surgical complications were uncommon. Overall, the most common complications were new cranial nerve palsy (n=7, 6.8%) (**Table 5**), followed by postoperative CSF leakage (n=6, 5.9%), intracranial infection (n=5, 4.9%), and ICA injury (n=2, 2.0%). New cranial nerve palsy consisted of oculomotor nerve and abducens nerve palsy, with an incidence of 3.9% and 2.9%, respectively. For the two patients with ICA injury, the injured ICA was completely clipped in one patient, and the ICA wall was electrocoagulated in the other patient. During the follow-up period, both patients underwent cerebral angiography; no pseudoaneurysm formation was found, and no obvious symptoms were observed. CSF leakage occurred in 6 patients and was managed by lumbar puncture catheter drainage in 2 patients and endoscopic repair surgery in the remaining 4 cases. Of the 5 patients with intracranial infection, 4 patients recovered through the use of antibiotics and lumbar puncture catheter drainage, and the remaining 1 patient died of infection.

**Table 5:** Complications of cranial nerve palsy.

	Postoperative			Improvement during follow-up		
	Total	Primary	Recurrent	Total	Primary	Recurrent
**CN III**	4	1	3	2	1	1
**CN IV**	0	0	0	0	0	0
**CN V**	0	0	0	0	0	0
**CN VI**	3	1	2	1	1	0

CN III, oculomotor nerve, CN IV, trochlear nerve, CN V, trigeminal nerve, CN VI, abducens nerve.

### Endocrinological Outcomes

Of the 102 patients, 40 cases (39.2%) were classified as functional PAs, and hormone remission was seen in 29 of these 40 patients (72.5%), postoperatively. Statistical analysis showed no statistical differences between endocrine outcomes and tumor size, tumor recurrence, or extent of CS lesion (p>0.05). However, for the entire tumor, patients with GTR had better endocrine outcomes than those with incomplete resection, and the difference was statistically significant (p=0.028). These results are shown in **Table 6**.

**Table 6:** Endocrine outcomes correlated with multiple potential variables.

Variables	Hormone remission	Hormone remission	P
	Yes	No	Value
**Size of tumor**			
Giant PA	13 (76.5)	4 (23.5)	
Macroadenoma	16 (69.6)	7 (30.4)	0.629
**Volume of tumor(V, cm3)**			
V<15	6 (75.0)	2 (25.0)	
15≤V ≤ 30	11 (78.6)	3 (21.4)	
>30	12(66.7)	6 (33.3)	0.744
**Extent of resection**			
GTR	16 (88.9)	2 (11.1)	
STR	11 (68.7)	5 (31.3)	
PTR	2 (33.3)	4 (66.7)	**0.028**
**Extent of CS resection**			
GTR	18 (75.0)	6 (25.0)	
STR	10 (71.4)	4 (28.6)	
PTR	1 (50.0)	1 (50.0)	0.744
**Tumor characteristics**			
Primary	19 (76.0)	6 (24.0)	
Recurrent	10 (66.7)	5 (33.3)	0.522

GTR, gross total resection; STR, subtotal tumor resection; PTR, partial tumor resection; CS, cavernous sinus. In bold: represent statistical significance.

### Visual Outcomes

Preoperative visual dysfunction was found in 38 patients, of which 28 patients (73.7%) improved postoperatively. For the entire tumor, the visual improvement rate was 90.9% in the GTR group, compared with 63.6% in the STR group and 20.0% in the PTR group. The difference between the three groups was statistically significant (p=0.003). However, preoperative visual function, extent of resection of CS lesion, tumor size, tumor volume, tumor recurrence or tumor consistency were not significantly associated with postoperative visual outcomes (p>0.05). These results are shown in **Table 7**.

**Table 7:** Visual outcomes correlated with multiple potential variables.

Variables	Improved	Unchanged	P value
**Preoperative visual symptoms**		
Unilateral	17(70.8)	7(29.2)	
Bilateral	11(78.6)	3(21.4)	0.601
**Size of tumor**			
Giant PA	19(79.2)	5(20.8)	
Macroadenoma	9(64.3)	5(35.7)	0.315
**Volume of tumor(V, cm^3^)**			
V<15	12(80.0)	3(20.0)	
15≤V ≤ 30	9(69.2)	4(31.8)	
>30	7(70.0)	3(30.0)	0.774
**Firm consistency **			
Yes	9(60.0)	6(40.0)	
No	19(82.6)	4(17.4)	0.122
**Extent of resection**			
GTR	20(90.9)	2(9.1)	
STR	7(63.6)	4(36.4)	
PTR	1(20.0)	4(80.0)	**0.003**
**Extent of CS resection**			
GTR	20(83.3)	4(16.7)	
STR	7(58.3)	5(41.7)	
PTR	1(50.0)	1(50.0)	0.203
**Tumor characteristics**			
Primary	18(85.7)	3(14.3)	
Recurrent	10(58.8)	7(41.2)	0.061

PA, pituitary adenoma; GTR, gross tumor resection; STR, subtotal tumor resection; PTR, partial tumor resection. In bold: represent statistical significance.

### Follow-Up Results

The follow-up period was 7-87 months, with an average of 46 months. Postoperatively, of the 30 patients with incomplete tumor resection, 5 patients with functional PAs received medical treatment and 12 patients (1 patient with functional PA and 11 patients with non-functional PAs) received radiotherapy. Among the 7 patients with postoperative new cranial nerve palsy, improvement was observed in 3 patients. For the 6 patients with preoperative cranial nerve palsy, improvement was seen in 4 patients. Six patients, including four patients with PTR, one with STR and one with GTR, experienced tumor recurrence and accepted reoperation. The follow-up methods were conducted through outpatient visits and telephone calls.

## Discussion

### Outcomes of Aggressive Tumor Resection

#### Extent of Resection

This study outlines the surgical outcomes and experiences of 102 patients with grade 4 PAs who underwent endoscopic transsphenoidal surgery. To date, this is the largest study conducted on grade 4 PAs, and is the first study to specifically focus on functional and non-functional grade 4 PAs. One of the study’s strengths is that all of the surgeries in this study were performed by the same surgeon, eliminating variability and rendering more credibility to the study results. Management of the intracavernous ICA contributes to some of the challenges surrounding the surgical removal of grade 4 PAs. To address this, individual spatial relationships among the ICA, the CS tumor, and various compartments of the CS were evaluated in detail preoperatively, and different CS approaches were adopted, accordingly. For example, when the horizontal ICA was superior to the tumor, the anterior wall of the CS was opened, and then an anteroinferior approach was used to remove the tumor from within the anteroinferior compartment, while a lateral-superior approach was used to remove the tumor from within the lateral compartment. This study represents the first time that these methods were carried out for grade 4 PAs. In this study, an aggressive resection strategy was adopted, not only for tumors in the medial CS, but also for tumors in the lateral CS. This approach differs from the previous surgical resection strategies of Woodworth et al. (18) and Toda et al. (19). Their surgical concept included using an aggressive strategy for tumors in the medial CS and a conservative strategy for tumors in the lateral CS. Both reported GTR rates for grade 4 PAs of no more than 10%. However, our study reported a GTR rate of 70.6%, which is significantly higher than the GTR rates (from 0 to 53.8%) reported in the previous literature (19–27) (**Table 8**). Of note, while we adopted an aggressive tumor resection strategy to obtain a considerable GTR rate, the incidence of surgical complications was still within an acceptable range. This indicates the feasibility and efficacy of aggressive resection strategy *via* transsphenoidal endoscopic surgery for grade 4 PAs.

**Table 8:** Comparison with similar studies of pituitary adenomas involving cavernous sinus published in the last decade.

Author	Year	No. of cases	Grade 1	Grade 2	Grade 3	Grade 4 (GTR %)	GTR (%)	Mean follow-up(m)
**Ceylan et al.** (20)	2010	19	0	0	9	10 (NR)	63.2	26
**Zhao et al.** (21)	2010	61	0	0	21	40 (47.5)	62.0	38
**Paluzzi et al.** (22)	2013	403	105	142	81	75 (0.0)	64.1	NR
**Taniguchi et al.** (23)	2015	25	0	0	23	2 (0.0)	56.0	36
**Ferreli et al.** (27)	2015	56	0	0	28	28 (17.8)	30.3	61
**Bao et al.** (24)	2015	52	0	0	13	39 (53.8)	63.5	24
**Kalinin et al.** (25)	2016	97	11	22	23	41 (NR)	50.5	NR
**Toda et al.** (19)	2018	30	0	0	0	30 (10.0)	10.0	NR
**Kosugi et al.** (26)	2019	23	0	0	7	16 (6.3)	8.7	NR
**Total**		766	116	164	205	281		
**Present study**	2020	102	0	0	0	102 (70.6)	70.6	46

NR, not reported; GTR, gross total resection.

#### Factors Influencing the Extent of Resection

The factors that influence the extent of resection for grade 4 PAs are unclear, because no previous study has ever specifically examined these potential factors. Any previous studies that did report on potential factors influencing the extent of resection included common PAs without CS invasion, or invasive PAs with varying extents of CS involvement. Chabot et al. (28) found that factors influencing the extent of resection included tumor recurrence, preoperative hormone replacement therapy, and Knosp grade. Bao et al. (24) also found that tumor recurrence and high Knosp grade were adverse factors affecting the extent of resection. In our study, we analyzed various potential factors, including tumor recurrence, tumor size, tumor volume, and tumor consistency, and found that tumor recurrence and consistency affected the extent of resection, while the other factors did not. In addition, we also found that there was no statistical correlation between the extent of tumor resection and the occurrence of CSF leakage and postoperative cranial nerve palsy (P >0.05).

#### Visual Outcome

In a 2012 systematic review by Komotar et al. (29), those patients who underwent transsphenoidal microscopic approach showed that the rate of visual improvement after PA surgery was 34.8%. Kalinin et al. (25) reported on a transsphenoidal endoscopic study of 97 patients with PAs invading CS in 2016, and vision improvement was achieved in 41.4% of cases. In a study of 39 patients with transsphenoidal endoscopic surgery, Chabot et al. (28) reported that 31 had CS invasion and the improvement rates of visual acuity and visual field were 73.9% and 72.4%, respectively. In our study, of the 38 patients who had visual impairment before surgery, visual improvement was achieved in 28 patients (73.7%), postoperatively. The visual results in our study were better than those reported for transsphenoidal microscopy, and this is broadly consistent with the results of previously published endoscopic studies. The superior visual outcomes of endoscopic endonasal surgery reflect the ability of the endoscope to visualize and protect the optic apparatus and its blood supply in the subarachnoid space, which these tumors can sometimes invade. In our study, patients with a total resection were more likely to have visual improvement than those with incomplete resection.

#### Endocrine Outcome

In addition to removal of the tumor, hormone remission is an important indicator of surgical outcome for functional PAs. Hofstetter et al. (30) reviewed 86 consecutive functional PAs (18 cases with CS invasion and 68 cases without CS invasion) by transsphenoidal endoscopy and the overall hormone remission rate was 60%. Bao et al. (24) reported a study of 52 cases of PAs that had invaded the CS, including 25 cases of functional PAs. Among the functional PAs, the hormone remission rate was 76%. In our study, among the 40 cases of functional PAs, the rate of hormone remission was 72.5%, which is consistent with previous studies. Patients with GTR of the entire tumor were more likely to experience hormone remission than those with incomplete resection (P<0.05). Of note, in our study, the endocrine outcomes were difficult to compare with previous endoscopic studies, which included tumors that did not invade the CS or different extents of CS invasion, whereas our study only included grade 4 PAs.

PRL-secreting adenomas with CS invasion present a significant challenge to pharmacologic treatment. In our study, indications for surgery mainly included drug-resistant tumors and patients who experienced drug intolerance due to side effects. Residual tumors in the CS can continue to cause endocrinological symptoms, and tumors with CS invasion are more likely to develop drug resistance. In some patients, residual CS lesions may remain even after continued medical treatment (31, 32).

#### Tumor Recurrence

PAs are benign tumors, although they often invade surrounding structures such as the CS and the sphenoid sinus. For benign tumors, patients have a good prognosis if the tumor can be completely removed; on the contrary, incomplete tumor resection may result in tumor recurrence. Prior studies have reported that incomplete tumor resection leads to a high recurrence rate, as high as 40-70% (33, 34). In contrast, the recurrence rate after GTR is significantly lower, with a 6-21% recurrence rate reported in the literature (33, 35). Notably, these studies include both invasive and non-invasive PAs, not just PAs invading the CS. Recently, Hwang et al. reported that up to 25% of cases with severe CS invasion recurred after total resection or near-total resection *via* a transsphenoidal endoscopic approach with long-term follow-up (11). In our study, 6 patients (5.9%), including 4 patients with PTR, 1 with STR and 1 with GTR, experienced tumor recurrence. Compared with the results reported in previous literature, the tumor recurrence rate in our study was relatively low. It is more difficult to achieve GTR in recurrent tumors, due to the formation of tissue adhesion and scar tissue, and changes that can occur in the normal anatomical structure after the initial operation. Additionally, the possibility of intraoperative injury to the ICA and surrounding cranial nerves is significantly increased (36). This was also confirmed in our study. Therefore, it is particularly important to completely remove the tumor during the first operation.

### The Safety of Aggressive Tumor Resection

#### Complications—ICA Injury

ICA injury is the most serious complication of transsphenoidal endoscopic surgery and is the main obstacle to GTR within the CS. Because of this, many tumors within the CS are not aggressively removed. In practice, however, it is not impossible to avoid ICA injury. Accumulated evidence has shown that the incidence of ICA injury by the transsphenoidal approach to PAs is low, reportedly 0-1.6% (22, 37–39). However, these studies include PAs with or without CS invasion. In one study, Raithatha et al. reported 41 cases of PAs involving CS that utilized transsphenoidal endoscopic surgery, and the incidence of ICA injury was 2.4% (40). In our study, among the 102 cases of grade 4 PAs, ICA injury occurred in 2 patients (1.9%). Given the severity of risk with CS invasion, the incidence of ICA injury in our study appears to be favorable, compared with what has been previously reported. In our experience, the risk of ICA injury can be reduced by the following: 1) Performing a preoperative cerebral angiography balloon occlusion test to evaluate the compensatory capacity of the contralateral ICA in case of ipsilateral ICA injury; 2) Utilizing intraoperative navigation and doppler ultrasonography to assist in determining the position of the ICA in advance; 3) Exposing the proximal end of the ICA before entering the CS to remove the tumor; and 4) Using three-dimensional MRI to individually analyze the spatial relationship between the ICA and the CS tumor, and further, to determine the size of each compartment in the CS. After following these steps, and, according to the individual spatial relationship, it is possible to then decide the point of entry to the CS and the type of surgical approach to take within the CS. If the above four objectives are achieved, and intraoperative ICA injury still occurs, then it is imperative to restore it intraoperatively in the best way possible. Once injury occurs to the ICA, various methods, such as electrocoagulation of the vessel wall, clipping the proximal end of the ICA with an aneurysm clamp, or interventional therapy, can be adopted to manage the injury according to the extent of injury. For the 2 patients with ICA injury in our study, the injured ICA was completely clipped in one patient, and the ICA wall was electrocoagulated in the other patient. During the follow-up period, cerebral angiography was performed on the 2 patients, and for both, no pseudoaneurysm formation occurred, and no obvious symptoms were observed. Of note, both of these patients with intraoperative ICA injury had undergone previous operations for PAs. This also further demonstrates the importance of GTR at the first operation.

#### Complications—Cranial Nerve Palsy

Cranial nerve palsy is a common surgical complication of PAs with CS invasion, especially in the transcranial microscopic resection of CS tumors, because in traditional craniotomy, the CS is entered from the lateral wall of the CS, which is the exact location of multiple cranial nerves. In current transsphenoidal endoscopic surgical procedures, the CS is entered through the medial or anterior wall of the CS, which has no nerve structures. Therefore, the incidence of cranial nerve palsy is low by transsphenoidal endoscopy for the removal of PAs from the CS. Toda et al. reported a study of 30 patients who underwent transsphenoidal endoscopic surgery; the GTR rate of grade 4 PAs was 10% and the complication rate of cranial nerve palsy was 6.7% (19). Similarly, another transsphenoidal endoscopic study of 52 patients included 13 patients with grade 3 PAs and 39 patients with grade 4 PAs, and the incidence of cranial nerve palsy was 9.6% (24). These studies both show that cranial nerve palsy occurred mainly in the oculomotor and abducens nerves. In contrast, trochlear and trigeminal nerve palsy rarely occurred. In our study, new postoperative cranial nerve palsy was found in 7 cases (6.8%), including 3 cases of abducens nerve palsy and 4 cases of oculomotor nerve palsy, which is consistent with the findings of previous literature. During the follow-up, improvement was observed in 3 of the 7 patients, including 1 case with abducens nerve palsy and 2 cases with oculomotor nerve palsy. As the oculomotor nerve is located in the oculomotor nerve triangle of the CS, damage to the oculomotor nerve can occur when removing the tumor from this area, especially a tumor that is invading the temporal lobe through the oculomotor nerve triangle. Of note, the oculomotor nerve triangle is often referred to as the blind area of transsphenoidal endoscopic surgery (41). Unlike the oculomotor nerve, the abducens nerve is not located on the lateral wall of the CS, but rather within the space of the CS and has adhesion connections with the ICA (12). During tumor resection, it is necessary to be familiar with the shape of the abducens nerve in the CS and to sharply separate adhesions from the ICA to reduce the incidence of injury to the abducens nerve. In our study, cranial nerve palsy occurred in 2 of the 60 patients (3.3%) with primary PAs, compared with 5 of the 42 patients (11.9%) with recurrent PAs. In order to avoid cranial nerve palsy for PAs with CS invasion, especially for recurrent PAs, it is necessary to monitor craniocerebral nerves, such as the oculomotor nerve and the abducens nerve, by means of intraoperative electrophysiological detection of extraocular muscle movement (42).

### Reasons for Aggressive Removal of Grade 4 PAs

In our study, the GTR rate of Knosp grade 4 PAs was significantly higher than any previously published study. Additionally, patients maintained favorable visual function and endocrine outcomes, and had low recurrence rates. Far more importantly, for an experienced endoscopist, such an aggressive tumor resection strategy did not significantly increase the incidence of complications. This favorable surgical outcome is due to the recent significant improvement in the understanding of Knosp grade 4 PAs. A preoperative analysis of the individual spatial relationship among the ICA, the CS tumor, and various compartments of the CS, coupled with intraoperative assistive techniques such as neuronavigation, doppler ultrasonography and cranial nerve monitoring, have enabled us to aggressively pursue GTR. However, it is important to emphasize that a successful procedure requires extensive experience in transsphenoidal endoscopic surgery.

## Conclusions

For experienced neuroendoscopists, an aggressive tumor resection strategy *via* transsphenoidal endoscopic surgery may be an effective and safe option for Knosp grade 4 PAs.

## Data Availability Statement

The original contributions presented in the study are included in the article/supplementary material. Further inquiries can be directed to the corresponding author.

## Ethics Statement

The studies involving human participants were reviewed and approved by the Institutional Ethics Committee of the First Affiliated Hospital of Nanchang University. The patients/participants provided their written informed consent to participate in this study.

## Author Contributions

Conception and design: TO and TH. Acquisition of data: JL, FZ, and LX. Analysis and interpretation of data: NZ, SX, and BW. Drafting the article: TO. Critically revising the article: DZ and BT. Reviewed submitted version of manuscript: TH. Approved the final version of the manuscript on behalf of all authors: TH. Statistical analysis: SX. Study supervision: TH, DZ, and ML. All authors contributed to the article and approved the submitted version.

## Funding

The present study was supported by the National Natural Science Foundation of China (grant no. 81760447; grant no. 81960247, grant no. 82060246), Project of Science and Technology Department of Jiangxi Province (grant no. 20192BBG70026, grant no. S2019QNJJB1056), and Jiangxi Provincial Education Department Project (grant no. GJJ180054; grant no. GJJ180116).

## Conflict of Interest

The authors declare that the research was conducted in the absence of any commercial or financial relationships that could be construed as a potential conflict of interest.
